# Is it reasonable to ignore vitamin D status for musculoskeletal health?

**DOI:** 10.12703/r/9-19

**Published:** 2020-12-03

**Authors:** Rebecca S Mason, Mark S Rybchyn, Tara C Brennan-Speranza, David R Fraser

**Affiliations:** 1 Physiology, Bosch Institute, School of Medical Sciences, Faculty of Medicine and Health, University of Sydney, Sydney, NSW, 2006, Australia; 2 School of Public Health, Faculty of Medicine and Health, University of Sydney, Sydney, NSW, 2006, Australia; 3 Sydney School of Veterinary Science, Faculty of Science, The University of Sydney, NSW 2006, Australia

**Keywords:** Vitamin D status, 25-hydroxyvitamin D, bone, muscle, parathyroid hormone, calcium, phosphate

## Abstract

Severe vitamin D deficiency—25-hydroxyvitamin D (25OHD) concentrations below around 25–30 nmol/L—may lead to growth plate disorganization and mineralization abnormalities in children (rickets) and mineralization defects throughout the skeleton (osteomalacia) and proximal muscle weakness. Both problems are reversed with vitamin D treatment. Apart from this musculoskeletal dysfunction at very low vitamin D levels, there is apparent inconsistency in the available data about whether concentrations of 25OHD below around 50 nmol/L cause muscle function impairment and increase the risk of fracture. This narrative review provides evidence to support the contention that improving vitamin D status, up to around 50 nmol/L, plays a small causal role in optimizing bone and muscle function as well as reducing overall mortality.

## Introduction

Over the past few years, one might be forgiven for holding the impression that vitamin D status is of little consequence for musculoskeletal function or much else^1–4^. This impression would be in distinct contrast to the view prevalent around 10 years ago that vitamin D is critical for almost every physiological function^5^. What follows is not a systematic review but an attempt to examine the role of the vitamin D system in bone and muscle function in light of older as well as more recent evidence.

## Physiology of vitamin D

Although mistakenly called a vitamin, vitamin D is really obtained by the action of ultraviolet light on skin, which opens the B-ring of 7-dehydrocholesterol to produce pre-vitamin D. At body temperature, pre-vitamin D converts to vitamin D3, also known as cholecalciferol^6^. Vitamin D3 or the fungal or yeast-derived vitamin D2/ergocalciferol^7^ may also be ingested through diet, though most unfortified foods contain very little vitamin D^8,9^. Vitamin D is converted in the liver, primarily, into 25-hydroxyvitamin D (25OHD), the major circulating metabolite with a long half-life^10^, which is in turn converted in the kidney, predominantly, into the active hormone 1,25-dihydroxyvitamin D—1,25(OH)2D, also known as calcitriol—which appears in the circulation. Both 25-hydroxylation and 1α-hydroxylation also occur in many other tissues but with the resultant hormone mainly available for use only by local cells^11,12^. Vitamin D status is measured by the concentration of the major circulating vitamin D metabolite, 25OHD, and not by concentrations of vitamin D itself or the hormone made from vitamin D, 1,25(OH)2D^13–15^.

There is little doubt that vitamin D plays a key role, along with calcium intake, in underpinning bone and muscle function. At blood concentrations of 25OHD below around 30 nmol/L, there is a risk of developing rickets (impaired growth plate dynamics), osteomalacia (undermineralization of bone matrix/osteoid), and muscle weakness, particularly affecting proximal muscles^15,16^. All of these signs are ameliorated by treatment with vitamin D^15,16^. Severe calcium deficiency can also cause rickets^17^. 1,25(OH)2D increases active intestinal absorption of calcium, provided there is ingested calcium to absorb. Calcium is not abundant in the diets of land-dwelling animals but is required for mineralization of new or replacement bone matrix and is important for many signaling processes in cells, including muscle contraction and relaxation as well as skeletal muscle development, growth, and regeneration^18^. Because of obligatory losses of calcium in urine and other waste, maintenance of calcium homeostasis is a constant issue for humans and other land-dwelling vertebrates^19^.

Without adequate vitamin D and/or calcium, parathyroid hormone (PTH) secretion increases. While PTH drives renal production of 1,25(OH)2D, reduces urinary calcium losses, and mobilizes bone resorption as an alternative source of calcium to maintain blood and extracellular calcium concentrations, it also drives urinary phosphorus excretion, reducing available phosphate. It is the reduction in phosphate, in particular, which appears to underpin the growth plate disorganization, in part due to impaired chondrocyte apoptosis^20^, that manifests as rickets. These actions of PTH may not be enough to maintain serum calcium concentrations under the circumstances. Impaired mineralization appears to be mostly a function of inadequate calcium and phosphorus availability, as these are the key minerals which make up the hydroxyapatite crystal of bone mineral. The relative importance of direct actions of 1,25(OH)2D in bone, especially considering that it is produced locally in this tissue^15^, is unclear, but two studies of calcium-deficiency rickets reported that vitamin D and calcium supplementation improved rickets in a larger proportion of children than calcium alone^21^, despite the already high gut calcium absorption rates in the children at baseline^17^.

## Vitamin D and muscle function

The underlying pathophysiology of muscle weakness in vitamin D deficiency is also not settled^22^. Vitamin D compounds affect skeletal muscle development and regeneration^23,24^. Whether this is direct or indirect is unclear, but there is some evidence that phosphorus deficiency may, at least in part, contribute^25^. Hypophosphatemic individuals display proximal muscle weakness^26^. Secondary hyperparathyroidism has also been postulated as a mechanism, since proximal muscle weakness is a recognized feature of primary hyperparathyroidism^27^. Other mechanisms have also been proposed. Impaired calcium signaling or changes in calcium fluxes in muscle cells contribute to the mechanism of muscle weakness in vitamin D deficiency^16,28–30^, potentially through impairment of calcium uptake into the endoplasmic/sarcoplasmic reticulum. Reductions in sarcoplasmic/endoplasmic reticulum ATPase (SERCA) proteins and calbindin have been reported in mice with myocyte knockout of the vitamin D receptor (VDR)^31^. Vitamin D treatment also improved mitochondrial oxidative phosphorylation in skeletal muscle in patients with severe vitamin D deficiency^32^. It is difficult to explain rapid subjective improvement of muscle weakness in severely vitamin D-deficient people, within 24 hours of an oral dose of vitamin D, in terms of correction of secondary hyperparathyroidism and hypophosphatemia.

The question of how important direct actions of 1,25(OH)2D or other vitamin D metabolites are for muscle function remains unanswered. Despite some controversy about whether VDRs are present in mature fresh muscle^33,34^, the current evidence indicates that they are found in mouse and human muscle tissue^35–38^, although at very much reduced levels compared with VDR levels in the intestine^35^. Mice with myocyte knockout of the VDR have low muscle mass and show reduced voluntary activity^31,39^, indicating at least a role for the VDR in muscle development. In a recent systematic review of genetic associations with aging muscle, polymorphisms in *VDR* were featured, with the highest number of studies carried out on this gene^40^, though even that review did not include all possible papers on the subject. As frequently noted in the literature, different studies have found different associations with particular polymorphisms of the VDR. In general, muscle function in older populations, variously measured, has been linked mostly to polymorphisms in Fok1 and Bsm1. The Fok1 site in exon 2 affects the translation initiation site^41^. The “f” allele denotes a start site for the VDR that results in a full-length VDR, whereas the “F” allele, which appears to be more transcriptionally active, codes for a later start site and a protein that is three amino acids shorter^41^. Most studies indicate a link between the presence of the “F” allele and worse muscle performance and/or sarcopenia^42–47^, but other studies showed no relationship^48^ or even a reverse association^49^. Polymorphisms in the Bsm1 allele in the 3'-untranslated region appear to affect mRNA stability of VDR^50,51^. The associations between either allele of Bsm1 and muscle strength/sarcopenia seem weaker and more variable than those for Fok1^40,45,47,48^. These studies were all carried out in older individuals with all the limitations of that approach. One study of 700 primary school children in Hungary found a strong association between highly heritable hand grip strength in both dominant and non-dominant hands with the *A1012G* (rs45160335) polymorphism in the 1e promoter region at the 5' part of the gene^52^. The presence of VDR in muscle cells has recently been shown to be critical for mitochondrial function^53^, and numerous VDR promoter elements in mitochondrial DNA have been described^54^. Observational studies indicate that muscle VDRs decrease with age^55^ and have been reported to increase with modest vitamin D supplementation in older women^56^.

Whether the problems of rickets and/or osteomalacia and proximal muscle weakness are entirely due to abnormal calcium or phosphate homeostasis has been examined in pre-clinical models. In mouse models, the rickets, osteomalacia, muscle weakness, and secondary hyperparathyroidism observed in mice with global knockout of the VDR is largely, if not entirely^57^, reversed by feeding the mice a rescue diet of increased calcium and phosphorus, together with high lactose, which appears to facilitate calcium absorption^58^, or by engineering VDR expression just in the intestine^59^. Apart from rickets and osteomalacia, high PTH concentrations, which may occur secondary to vitamin D deficiency, are associated with increased cortical bone porosity and associated increased risk of fracture^60,61^. In human patients with VDR mutations that impair function, calcium infusions coupled with high-calcium diets appear to allow for growth and mineralization through childhood^62^. For reasons that are not clear, once these affected patients reach maturity and so are only replacing bone being remodeled and not growing a skeleton, very high calcium intakes are no longer required, as calcium absorption is increased by non-VDR mechanisms, thus relieving the secondary hyperparathyroidism and associated hypophosphatemia^62^. These patients seem to have relatively normal bone in adulthood and are able to lead a relatively normal life, including having children^62,63^.

These observations tend to support the proposal that the key musculoskeletal action of the vitamin D system is to enhance gut calcium absorption and so prevent the secondary hyperparathyroidism and resultant urinary phosphorus losses^15^.

Proximal muscle weakness is a hallmark of vitamin D deficiency at all ages^30^. Vitamin D status is a predictor of muscle function across adult age groups^64^, particularly in older individuals^65^, and may even predict loss of muscle mass and falls in older people^66,67^. Even so, since there is evidence that muscle, like other tissues, expresses both 25-hydroxylase and 1α-hydroxylase^68,69^, an association between vitamin D status, as measured by circulating 25(OH)D, and muscle function may not be apparent under some circumstances. Declines in muscle function, particularly in older people, are observed below 25OHD concentrations of 40–60 nmol/L^64,65^. Older studies in students showed improvements in muscle performance after exposure to ultraviolet light^70^. In athletes, who, as a group, have a rather low vitamin D status^71^, supplementation with vitamin D has produced variable responses, even in studies from the same group^72,73^. A recent meta-analysis showed no overall improvement in athletic performance with vitamin D supplements^74–76^. In non-athletes, vitamin D supplementation studies have also produced mixed results^74,77–79^ (reviewed in 16 and 80). Benefits of vitamin D supplementation have mainly been reported in people with severe vitamin D deficiency, such as veiled women with a mean 25OHD concentration around 7 nmol/L^81^, in studies where vitamin D was given with calcium^82^, where doses of 800–1,000 IU/day were used^83^, and in older individuals with baseline 25OHD concentration below 25 or 30 nmol/L^84–86^.

## Vitamin D and bone

In relation to bone density, based on large-scale observational data, there is a positive correlation between 25OHD concentrations and bone density. In two major studies, one in younger and older adults and the other in older people, bone mineral density increased with increasing 25OHD but reached a plateau at around 50–60 nmol/L^87,88^. The effects of higher vitamin D status on bone density are relatively small^87–89^, much smaller than the effects of age, for example^90^. In a number of randomized controlled trials (RCTs) of older individuals, supplementation with vitamin D alone has been reported to have no effect or a small positive effect on bone mineral density^91^, but vitamin D supplements with calcium had relatively small positive effects on bone mineral density^92,93^, with significant effects mostly seen in those with 25OHD levels below 30–40 nmol/L at baseline^94^.

Increased osteoid (unmineralized bone matrix) of >5% bone volume is a hallmark of severe vitamin D and/or calcium deficiency^95^ but was reported in only 10% of hip fracture patients with a 25OHD of <30 nmol/L^96^. One German study of post-mortem samples of blood and bone after acute trauma found no evidence of osteomalacia/excess osteoid (>5%) provided subjects had blood 25OHD concentrations above 50 nmol/L^97^. Interestingly, the taskforce for the Endocrine Society USA used the same data to support the case for a 75 nmol/L target for 25OHD by classifying abnormal osteoid at >2% rather than >5%^98^.

It might be argued from the above data that even moderate degrees of vitamin D deficiency should cause musculoskeletal dysfunction with increased risks of falls, due to muscle weakness and/or impaired coordination, and fractures. These associations have certainly been reported^15^. The risk of falls and fractures in young people is relatively low, so most RCTs tend to be done in older populations. If low vitamin D status increases the risk of falls and fractures, replacement of vitamin D in these moderately deficient subjects should reduce these events. But that is not the consistent outcome of RCTs.

Results of RCTs of vitamin D supplements, often with calcium, have been variable for falls^99–103^. Overall, though, these studies showed a modest, around 15%, reduction in the risk of falls^103^, or of fallers, mostly when supplements of more than 700 IU were given^99^ (this dose is likely to raise 25OHD above 50 nmol/L) along with adequate calcium intake or supplemental calcium^104^ and when the target population was in institutionalized care, with low vitamin D status^100^ or ambulatory 25OHD values below 50 nmol/L^101^.

A recent meta-analysis of RCTs of vitamin D supplements to reduce fracture risk reported that supplementation with vitamin D alone did not affect fracture incidence but supplementation with vitamin D and calcium reduced fractures significantly^105^. This has been reported before in a Cochrane review^106–108^. Even so, most of these meta-analyses (reviewed^1,15,109^) indicate an overall small reduction in fractures with vitamin D and calcium together, with more benefit seen in patients at higher risk of fracture and those given at least 800 IU of vitamin D a day. That dose of vitamin D would be expected to increase blood 25OHD concentration to above 50 nmol/L^110^. Although this small reduction in fracture risk of around 15% has been described as unimportant by some^1,111^, this is unlikely to be the view of most patients at risk or the health system that pays for the immediate and long-term costs.

These results, which overall indicate that supplementation with both vitamin D and calcium is needed to reduce fracture risk to at least a small extent and mainly in susceptible populations, are perhaps not entirely surprising. If the main effect of adequate vitamin D status is to increase the absorption of gut calcium, it ought to help if calcium intake is also adequate. In many older people, it is not. Analyses which specifically exclude those studies in which vitamin D and calcium were given are thus more likely to report outcomes of no effect from vitamin D supplementation^105^. Indeed, when high doses of vitamin D have been given intermittently, increased falls and fractures have been reported^112,113^. Then there are other considerations. Vitamin D has been described as a “threshold nutrient”^15^: if people have enough vitamin D (25OHD), giving more is not likely to make much difference. In many RCTs, the mean or median vitamin D status of participants, measured by 25(OH)D at baseline, is well above any reasonable threshold of 30 nmol/L^14,15^ or 50 nmol/L^107,114^. Some studies specify analysis of subgroups deemed low in vitamin D, but numbers are necessarily lower than in the main group, especially in studies in North America, where food fortification with vitamin D is extensive^8,9^.

## Vitamin D and mortality

The hormonal form of vitamin D, 1,25(OH)2D, when bound to its receptor, affects the transcription of hundreds of genes^115^. Not surprisingly, low vitamin D status, however defined, has been linked with increased risks of a range of health issues, such as increased inflammation, increased risk of some autoimmune conditions, increased risk of some cancers, poor sleep, poorer outcomes of pregnancy, increased risk of type 2 diabetes, and increased overall mortality. While there are problems with the design and/or implementation of many RCTs for vitamin D^116^, the frequently negative results for non-musculoskeletal outcomes have led to the argument that low vitamin D status is mainly a marker of poor health, which may indeed be the case^117^. Two recent meta-analyses of RCTs for the prevention of acute respiratory infection or reducing exacerbations of asthma requiring corticosteroid treatment found a small benefit overall with daily or weekly vitamin D supplements, but the benefit was most pronounced in individuals with 25OHD concentrations below 25 nmol/L at baseline^118,119^.

There is a clear association between low vitamin D status and increased age-adjusted mortality. Two observational studies involving a quarter of a million Danes and 15,000 people from the NHANES cohort showed an increased risk of death over 5–6 years in people with a baseline 25OHD level below approximately 50 nmol/L^120^ or 40–75 nmol/L depending on sex^121^. Elevated PTH levels, even within the normal range, are known to increase risk of mortality^120,122^. With some exceptions^1^, meta-analyses of RCTs of vitamin D mostly with calcium supplementation, which recorded mortality as a safety endpoint rather than the primary outcome, indicated that supplementation reduced mortality in the treatment groups in older individuals with a mean 25OHD level of approximately 37 nmol/L at baseline by 6–11%^123–125^.

## Issues with RCTs of vitamin D

There are many further caveats to the use of RCTs as a means of demonstrating causality in relation to the associations between vitamin D status and musculoskeletal or other health parameters. Most studies of vitamin D supplementation with or without calcium have used white Caucasian subjects. The target optimal 25OHD concentration to be achieved in non-whites is not as clear and may be lower in some groups^126^. Assays for 25OHD are relatively variable compared to other hormone assays^14,116,127^. It must also be kept in mind that some people with 25OHD concentrations below 30 nmol/L may have little evidence of biochemical or musculoskeletal abnormalities^2^. Furthermore, once 25OHD blood concentrations are above 25–30 nmol/L or so, the observed effects of vitamin D status on many functions are small compared with other more major factors, like sex steroids and age on bone mass, age and exercise on muscle mass, carcinogens or lifestyle factors and genetic influences on cancer and cardiovascular disease, and thus overall mortality.

If one looks at vitamin D status at various ages in a developed western country like Australia, some interesting patterns emerge. In a very large Australian Bureau of Statistics study of approximately 10,000 people from the electoral roll with 25OHD analysis by liquid chromatography mass spectroscopy, it was those aged 18–34 who had the highest prevalence of vitamin D deficiency^128^. At these ages, people spend a lot of time inside, for entertainment, raising children, and working. It seems younger children are less at risk of low vitamin D status, but there is more of a problem in the teen years. People in the early pre-retirement or retirement years, aged 60–74 years, also had better vitamin D levels, whether because of more supplementation awareness or more leisure time^128^. In the younger and middle years, when bone and muscle mass and function are relatively high, the risks of falls, fractures, or death are relatively low and very much less likely to be greatly affected by vitamin D status. But prudence suggests that it is probably better to maintain target vitamin D levels, even during this period of life, to help provide a better baseline for life in more advanced years^65^.

## Conclusions

There is fairly uniform evidence and agreement that 25OHD levels below 25–30 nmol/L are likely to be detrimental to musculoskeletal health, especially if calcium intake is not optimal. A potential unifying hypothesis is that low vitamin D status, especially with low calcium intake, results in a tendency to negative calcium balance and a secondary increase in PTH concentrations, which cause renal phosphate wasting and hypophosphatemia. The low available calcium, low phosphate, elevated PTH, and/or low vitamin D impair muscle function and repair, leading to proximal muscle weakness. The low phosphate interferes with chondrocyte maturation at the growth plate (rickets) and, together with relatively low calcium, reduces mineralization of osteoid (osteomalacia) while the increased PTH increases bone resorption, resulting in cortical bone porosity and increased fracture risk. If elevated PTH is part of the problem, it is not surprising that supplying both vitamin D and calcium diminishes the problem. At a younger age, none of this may matter greatly. Most interpretations of meta-analyses of RCTs in older individuals tend to support the proposal that improving vitamin D status, up to around 50 nmol/L, plays a small causal role in optimizing bone and muscle function as well as reducing overall mortality.
